# Structural and functional features of medium spiny neurons in the BACHDΔN17 mouse model of Huntington’s Disease

**DOI:** 10.1371/journal.pone.0234394

**Published:** 2020-06-23

**Authors:** Joseph Goodliffe, Anastasia Rubakovic, Wayne Chang, Dhruba Pathak, Jennifer Luebke

**Affiliations:** Department of Anatomy & Neurobiology, Boston University School of Medicine, Boston, Massachusetts, United States of America; University of South Alabama, UNITED STATES

## Abstract

In the BACHD mouse model of Huntington’s disease (HD), deletion of the N17 domain of the Huntingtin gene (BACHDΔN17, Q97) has been reported to lead to nuclear accumulation of mHTT and exacerbation of motor deficits, neuroinflammation and striatal atrophy (Gu et al., 2015). Here we characterized the effect of N17 deletion on dorsolateral striatal medium spiny neurons (MSNs) in BACHDΔN17 (Q97) and BACWTΔN17 (Q31) mice by comparing them to MSNs in wildtype (WT) mice. Mice were characterized on a series of motor tasks and subsequently whole cell patch clamp recordings with simultaneous biocytin filling of MSNs in in vitro striatal slices from these mice were used to comprehensively assess their physiological and morphological features. Key findings include that: Q97 mice exhibit impaired gait and righting reflexes but normal tail suspension reflexes and normal coats while Q31 mice do not differ from WT; intrinsic membrane and action potential properties are altered -but differentially so- in MSNs from Q97 and from Q31 mice; excitatory and inhibitory synaptic currents exhibit higher amplitudes in Q31 but not Q97 MSNs, while excitatory synaptic currents occur at lower frequency in Q97 than in WT and Q31 MSNs; there is a reduced total dendritic length in Q31 -but not Q97- MSNs compared to WT, while spine density and number did not differ in MSNs in the three groups. The findings that Q31 MSNs differed from Q97 and WT neurons with regard to some physiological features and structurally suggest a novel role of the N17 domain in the function of WT Htt. The motor phenotype seen in Q97 mice was less robust than that reported in an earlier study (Gu et al., 2015), and the alterations to MSN physiological properties were largely consistent with changes reported previously in a number of other mouse models of HD. Together this study indicates that N17 plays a role in the modulation of the properties of MSNs in both mHtt and WT-Htt mice, but does not markedly exacerbate HD-like pathogenesis in the BACHD model.

## Introduction

Huntington’s Disease (HD) is an autosomal dominant neurodegenerative disorder caused by CAG repeat expansion in exon 1 of the Huntingtin gene (Htt) [1]. The ubiquitous expression of the mutant Huntingtin protein (mHtt) results in the manifestation of psychiatric, behavioral, and motor symptoms including choreiform movements and bradykinesia [2–4]. Of these symptoms, sub-lethal dysfunction of striatal neurons likely leads to early-stage choreiform signs, while late-stage motor dysfunction or bradykinesia results from the death of striatal neurons [4–6]. Extensive neurodegeneration characteristic of HD occurs in numerous brain regions, including neocortex, thalamus, hippocampus, and striatum [7–10]. Typically, the striatum is dramatically affected, with the caudate and putamen showing extensive reductions in volume [10–12]. Medium spiny neurons (MSNs) of the striatum are the recipients of a majority of excitatory afferents entering the striatum and are selectively vulnerable in HD [11, 13]. As the most numerous neuron population within the striatum MSNs are the major contributors to the cortico-striatal-thalamocortical loops that regulate motor behavior and other cognitive processes; thus, dysfunction and death of MSNs critically compromise neural circuits in HD [13–15]. The MSN population is comprised of two sub-types -D1 and D2- which express the Dopamine receptor 1 and dopamine receptor 2, respectively and the outputs of these populations give rise to the Direct (D1) and Indirect (D2) pathways [16–19]. Post-mortem and PET evidence suggest that D1 and D2 populations are differentially affected as HD progresses and this has led to the belief that D2 MSN loss precedes D1 MSN loss; however, limitations in human studies have left a large gap in understanding the neuronal dysfunction that may precede and lead to neuronal death [20–27]. Mouse models have facilitated the investigation of the mechanisms which may underlie neuronal and motor dysfunction in HD. Widely employed models such as BACHD, R6/2, YAC128, and Q175 have contributed to our understanding of the pathophysiological consequences of mHtt expression in the striatum and other brain areas [28–34]. In these models, increased intrinsic excitability (associated with increased input resistance and reduced rheobase), reduction in excitatory postsynaptic current (EPSC) frequency and reductions in MSN dendritic spine density have been reported [28–34].

Recently, mouse models have been developed that enable more detailed dissection of the functional role of different domains of the Htt gene in HD pathogenesis, including the polyQ expansion, the polyproline domain, and the N-terminal 17 amino acid domain (N17). The polyQ expansion is critical for the aggregation of the mHtt protein, while the polyproline domain is important for various protein interactions [35, 36]. The N17 domain contributes to mHtt aggregation, but is also able to suppress aggregation through post-translational modifications at specific serine residues giving the domain a dual function in aggregation [37–41]. The N17 domain has other critical functions including cytoplasmic membrane association and the nuclear export of the Htt protein [42–46]. While the functions of the N17 domain have been well studied *in vitro*, few *in vivo* studies have investigated how the N17 domain influences pathophysiology in animal models of HD. In a zebrafish model of HD, the deletion of the N17 domain (ΔN17) results in the accelerated development of motor dysfunction, reduced brain weight, and nuclear aggregation of the ΔN17-mHtt protein [47]. In the BACHD mouse model, deletion of the N17 domain (BACHDΔN17, Q97) has been reported to result in motor deficits, including abnormal head movements reminiscent of chorea seen in HD, changes to field potential activity, striatal neuronal loss, nuclear accumulation of mHTT, and neuroinflammation [48]. Importantly, there were no reported effects of N17 deletion on wild-type Htt (BACWTΔN17, Q31) with regard to body weight, brain weight, motor function or nuclear Htt accumulation [48]. The initial report of exacerbated pathophysiology in BACHD mice lacking N17 led us to query whether deletion of the N17 domain leads to exacerbation of alterations in the structure and function of MSNs in this model. We thus characterized the effects of N17 deletion on electrophysiological and morphological properties of MSNs in the BACHDΔN17, BACWTΔN17, and wild type (WT) mice (hereafter referred to as Q97, Q31, and WT, respectively) that were also assessed with regard to motor function.

## Materials and methods

### Experimental subjects

Mice were obtained from Jackson laboratories at ~5–6 months-of-age and all experiments were performed on mice at 9 months-of-age. Mice were housed and handled according to animal care guidelines from the NIH *Guide for the Care and Use of Laboratory Animals* and *the U*.*S*. *Public Health Service Policy on Humane Care and Use of Laboratory Animals* and research procedures were approved by the Institutional Animal Care and Use Committee at Boston University School of Medicine. We employed Drd2-eGFP x Wild-Type (WT), Drd2-eGFP x BACHDΔN17 (Q97), and Drd2-eGFP x BACWTΔN17 (Q31) male and female mice maintained on a CD-1 background strain in these studies. A total of 10 WT (WT; 5 females, 5 males), 8 Drd2-eGFP x Q97ΔN17 (Q97) (4 females, 4 males; mean ± SD CAG length 99 ± 0), and 7 Drd2-eGFP x Q31ΔN17 (Q31) (5 males, 2 females; mean ± SD CAG length 33 ± 0) mice were used in these studies.

### Assessment of motor function

Mice were weighed and assessed for quality of their coat prior to being assessed on three different tests of motor function including: tail suspension, self-righting, and gait assessment [49]. Coat quality was rated on a scale of 0 (normal, shiny)- 4 (matted, unkempt, yellowing). During testing, mice were videotaped and subsequently videos were analyzed by three researchers blinded to gender and genotype. For tail suspension, mice were suspended by their tails, one third of the length away from the base of the tail. Mice were suspended and videotaped for 15 seconds. The tail suspension task was scored on a scale of 0 (normal splaying of toes and legs)- 4 (curled toes, both legs close to body) For assessment of self-righting ability, mice were placed onto their backs and then released. This was repeated twice and videotaped for 20 seconds each time. Self-righting was scored as 0 (mouse rights self under 5 seconds)- 4 (mouse takes more than 5 seconds to right itself). For assessment of gait, mice were placed in an 18 inch x 18 inch x 5-inch arena and videotaped for 60 seconds. Mice were assigned a gait score from 0 (normal limb movement, feet under body)- 4 (limbs splay when walking, unsteady) based on the scoring system previously reported [49]. Total score was obtained by summing the scores on tail suspension, self-righting, gait, and coat quality.

### Slice preparation

Mice were anesthetized with isoflurane and rapidly decapitated. Brains were rapidly removed into oxygenated ice-cold Ringer’s solution (concentrations in mM: 25 NaHCO_3_, 124 NaCl, 1 KCl, 2KH_2_PO_4_, 10 glucose, 2.5 CaCl_2_, 1.3 MgCl_2_, 5 ATP, pH 7.4). Brains were cut on a vibratome into 300 μm slices in ice cold oxygenated Ringer’s solution from the most rostral aspect to the most caudal extent of the striatum. Slices were equilibrated for a minimum of 1-hour in oxygenated room temperature Ringer’s solution and then positioned in submersion recording chambers (Harvard Apparatus) on Nikon E600 IR-DIC microscopes [50–53]. Slices were continuously perfused with RT Ringer’s solution (2–2.5 ml/min). The dorsolateral striatum was defined by dividing the striatum into quadrants along the dorsal/ventral and medial/lateral axes. The quadrant that was lateral to the midline and dorsal to the ventral surface of the slice was considered to be the dorsolateral striatum [53–55]. D1 (eGFP-) and D2 (eGFP+) MSNs in the dorsolateral striatum were provisionally identified at the time of recordings by visualizing MSN somata under infrared-differential interference contrast (IR-DIC) optics then switching to epifluorescence optics to determine if the somata showed GFP signal [53, 55]. GFP expression was later confirmed using confocal microscopy (see Confocal imaging).

### Electrophysiology

Whole-cell patch clamp recordings were obtained from visually identified MSNs in the dorsolateral quadrant of the striatum [53, 55]. Electrodes were pulled on a Flaming and Brown horizontal pipette puller (model P87, Sutter Instrument) and filled with potassium methanesulfonate (KMS) internal solution, concentrations in mM as follows: (KCH_3_SO_3_ 122, MgCl_2_ 2, EGTA 5, NaHEPES 10, Na_2_ATP 5 and 1% biocytin). Electrodes in Ringer’s solution had a resistance of 4–6 MΩ. Electrophysiology data was obtained using PatchMaster software (HEKA Elektronik) and EPC-9/EPC-10 amplifiers (HEKA Elektronik).

#### Assessment of intrinsic membrane and action potential properties

Passive membrane properties (resting membrane potential–Vr-, input resistance–Rn- and membrane time constant –τ-) and action potential firing properties were assessed under current clamp [50–53]. Vr was measured as the voltage in the absence of current injection. A series of 200 ms or 2s hyperpolarizing and depolarizing current steps was applied for the rest of the measures. The voltage responses to each step were measured at steady state and plotted on a voltage-current graph: Rn was calculated as the slope of the best-fit line through the linear portion of the plot. Membrane time constant was measured by fitting a single exponential function to the membrane voltage response to the -10 pA hyperpolarizing step. Rheobase was determined with a 10 s depolarizing current dual ramp (0–100 pA, 0–200 pA; 3.03 kHz sampling frequency). Single AP properties, including threshold and amplitude, were measured on the second evoked AP in a 200 ms current-clamp series in which the current step elicited 3 or more action potentials. An expanded timescale and the linear measure tool were used in FitMaster analysis software (HEKA Elektronik). Finally, a series of 2 s hyperpolarizing and depolarizing steps (-200 to +450 pA, using 25 or 50 pA increments, 12.5kHz sampling frequency) was used to assess repetitive AP firing. Firing rate in response to current steps was analyzed with a generalized linear model, using the genotype, MSN type, rheobase, input resistance, injected current level and their respective interactions as independent variables.

#### Assessment of spontaneous excitatory postsynaptic currents

AMPA receptor-mediated spontaneous excitatory currents (sEPSCs) were recorded for 2 min at a holding potential of -80 mV (6.67 kHz sampling frequency). MiniAnalysis software (Synaptosoft) was used to assess synaptic current properties including: frequency, amplitude, area, time to rise and time to decay. For assessment of kinetics, the rise and decay of averaged traces were each fit to a single-exponential function. For all synaptic current measurements, the event detection threshold was set at the maximum root mean squared noise level (5 pA).

### Streptavidin-Alexa labeling of biocytin filled neurons

Following recordings, brain slices were sandwiched between filter paper in fixative (4% paraformaldehyde) overnight at 4°C. Next, slices were washed in 0.1 M phosphate buffered saline (PBS) 3x 5 minutes. Slices were then incubated in 0.1% Tx-100/PBS for 2 h at room temperature (RT), then incubated in Streptavidin-Alexa 568 (1:500, 0.1% Tx-100/PBS) for 2d at 4°C, followed by subsequent washes in 0.1 M PBS and stored in anti-freeze solution (30% glycerol, 30% ethylene glycol in 0.05 M phosphate buffer) [50–53].

### Confocal imaging

For verification of eGFP labeling of biocytin-filled cells, slices were placed in an inverted well slide and temporarily coverslipped for an initial imaging of the soma using a 40x oil immersion objective on a Leica SPE confocal microscope. Cell somata were scanned in their entirety in two channels, 568 and 488, to detect filled cells and the presence or absence of somal eGFP, respectively. Cells were classified as D2/eGFP+ if the 488 and 568 signals overlapped along the x-, y-, and z- planes, D1/eGFP- cells lacked eGFP signal in their soma. Brain slices that contained cells met rigid criteria for morphometric analysis (zero to minimal dendritic varicosities, high signal-to-noise ratio indicative of a well filled cell, few cut branches) were either mounted in Prolong Antifade (Life Technologies) after streptavidin-Alexa staining or used for immunohistochemistry. MSNs were scanned for dendritic morphometric analyses in their entirety using a Leica SPE confocal microscope with a 40x oil immersion objective obtaining a voxel size of 0.27 x 0.27 x 0.5 μm (as described previously [50,51]). Images were deconvolved with AutoQuant Software and 8-bit images imported into NeuronStudio for reconstruction and quantitative analysis. For assessment of spines, 3 dendrites from each cell were chosen for imaging of spines. Each dendrite was selected by dividing the entire dendritic arbor into equal thirds and selecting the dendrite that were located perpendicular to the z-plane. In order to obtain the necessary resolution for spine sub-typing, dendrites were imaged with a 63x oil emersion objective (1.4 NA) with a 2.5 zoom using a Leica SPE laser scanning confocal microscope. The resulting voxel size was 0.034 x 0.034 x 0.17 μm. Each dendrite was imaged in its entirety from the soma to the distal dendrite ending. Z-stacks were deconvolved using AutoQuant software and 8-bit images imported into Neurolucida 360 for reconstruction, spine sub-typing, and analysis. Spine sub-types were classified based on spine head diameter and spine head distance from the dendritic shaft. Spines were classified as: thin (diameter ≤ 0.6 μm), mushroom (diameter > 0.6 μm), stubby (spines lacking a neck), or filopodia (length > 3 μm) [50,52].

### Statistical analyses

Statistical analyses of empirical data were performed using GraphPad. For all data, Shapiro-Wilk test of normality were run. For data that passed the Shapiro-Wilk test, two-way ANOVAs were used to compare the three genotypes. Post hoc Tukey tests were performed to identify which group means differed significantly. For all data that failed Shapiro-Wilk test of normality, Kruskal-Wallis tests were used to compare the three genotypes followed by Dunn’s multiple comparison test to identify which group means differed significantly. All post-hoc p-values for significant findings are reported in figure legends.

## Results

### Decreased body weight of male Q97 mice and decreased brain weight in both male and female Q97 mice

**Fig 1:**
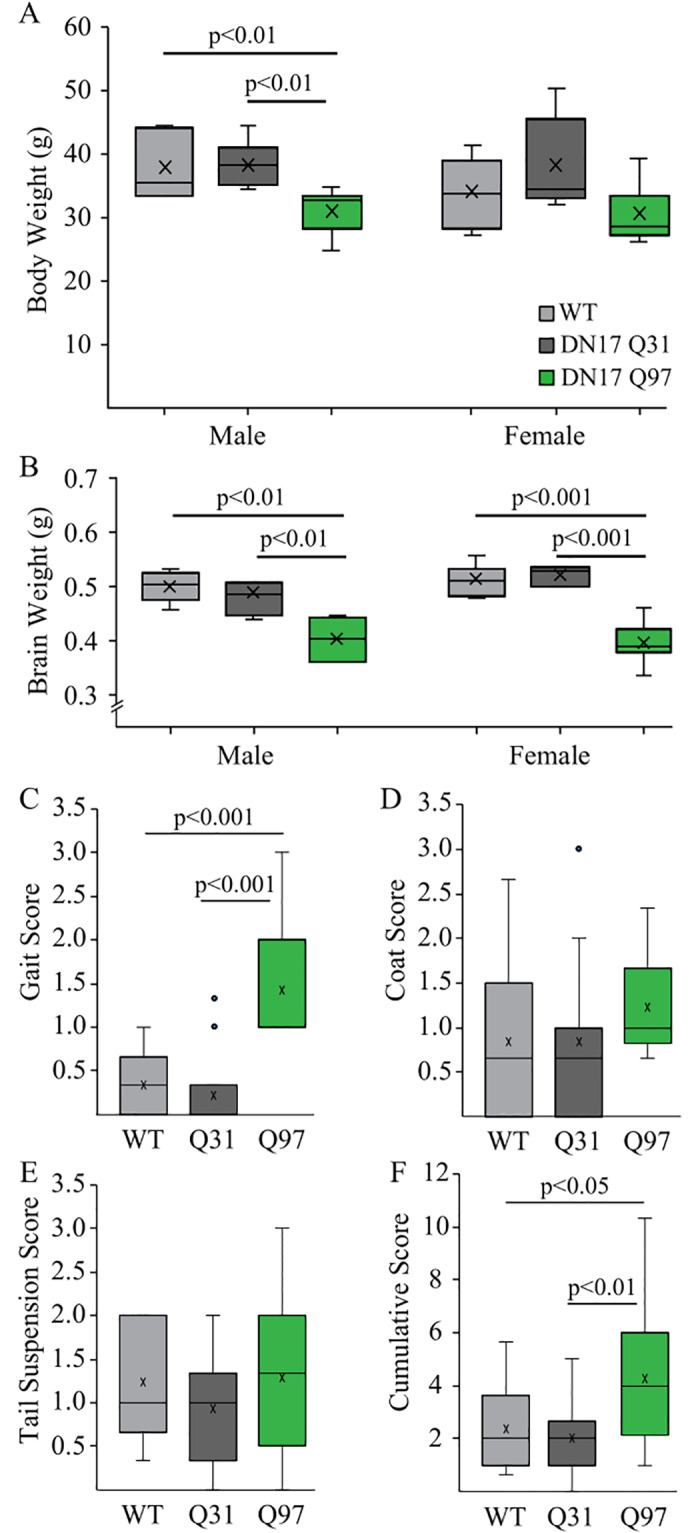
Body and brain weight and motor function in WT, Q31 and Q97 mice. (a) Male Q97 mice weighed significantly less than both Q31 and WT mice, while there was no difference between genotypes in female mice (Male: WT, n = 6; Q31, n = 10; Q97, n = 7; F_(2,22)_ = 8.66, p = 0.002; Female: WT, n = 7; Q31, n = 5; Q97, n = 7; F_(2,18)_ = 2.47, p = 0.116). (b) In both males and females, brain weight was significantly lower in Q97 compared to Q31 and WT subjects (which did not differ, Male: WT, n = 6; Q31, n = 10; Q97, n = 6; F_(2,21)_ = 9.45, p = 0.001; Female: WT, n = 7; Q31, n = 5; Q97, n = 7; F_(2,18)_ = 33.2, p<0.001;). (c) Q97 mice exhibited significantly impaired gait (WT, n = 11; Q31 = 15; Q97 = 11). (d) Coat quality did not differ between groups (WT, n = 13; Q31 = 15; Q97 = 14). (e) Tail suspension score did not differ between groups (WT, n = 13; Q31, n = 15; Q97, n = 14). (f) Cumulative score of animal performance in the motor tasks plus coat quality assessment revealed significant impairment in Q97 mice (WT, n = 13; Q31, n = 15; Q97, n = 14; p-values from *posthoc* Tukey tests).

Wild-type (WT), BACWTΔN17 (Q31), and BACHDΔN17 (Q97) mice were weighed at 9 months of age prior to sacrifice and preparation of striatal slices for whole-cell patch clamp experiments. Male Q97 mice weighed significantly less than both WT and Q31 males (WT, n = 6; Q31, n = 10; Q97, n = 7; F_(2,22)_ = 8.66, p = 0.002; Fig 1a). Body weight did not differ for female mice of the three genotypes (WT, n = 7; Q31, n = 5; Q97, n = 7; F_(2,18)_ = 2.47, p = 0.116; Fig 1a). Brain weight was significantly reduced in both male and female Q97 mice compared to WT and Q31 brains (Male: WT, n = 6; Q31, n = 10; Q97, n = 6; F_(2,21)_ = 9.45, p = 0.001; Female: WT, n = 7; Q31, n = 5; Q97, n = 7; F_(2,18)_ = 33.2, p<0.001; Fig 1b).

### Q97 mice exhibit impaired gait and righting reflex but normal tail suspension reflex and normal coat while Q31 mice do not differ from WT

Behavioral analysis of Q31 and Q97 mice have previously shown poor performance on rotarod tasks as early as 2 months of age and gait deficits at 8 months [48]. Here, we assessed all mice on a battery of motor tests which included: self-righting, gait, and tail suspension; in addition, we scored coat quality as described previously [49]. Scores did not differ significantly between male and female mice within each cohort so data from males and females were pooled for statistical analyses (F_(1,36)_ = 3.094, p = 0.0871). In the self-righting task, all WT and Q31 mice were able to right themselves in under 5 seconds (and thus received a score of 0), while some Q97 mice exhibited a delay of more than 5 seconds (WT = 0, Q31 = 0, Q97 = 0.9±0.2; Table 1). Q97 mice also exhibited gait deficits as evidenced by splayed and unsteady limb movements, compared to WT and Q31 mice (WT, n = 11; Q31, n = 15; Q97, n = 11; F_(2,36)_ = 22.87, p<0.001, Fig 1c). By contrast, coat quality and tail suspension scores did not differ between genotypes (Coat: F_(2,40)_ = 1.01, p = 0.37, Fig 1d; Tail Suspension: F_(2,41)_ = 0.96, p = 0.39, Fig 1e). The cumulative score of animal performance in the motor tasks plus coat quality assessment revealed significant impairment in Q97 mice due to gait and righting deficits (Overall: F_(2,41)_ = 5.76, p<0.01, Fig 1f).

**Table 1:** Behavioral analysis of WT, Q31, and Q97 animals reveal motor deficits in Q97 mice.

		Tail	Gait	Righting	Coat	Overall Score
WT	n	13	11	9	13	13
	avg	1.23	0.33	0.00	0.85	2.36
	stder	0.19	0.11	0.00	0.27	0.46
Q31	n	15	15	13	15	15
	avg	0.93	0.22	0.00	0.84	2.00
	stder	0.17	0.11	0.00	0.23	0.41
Q97	n	14	11	11	13	14
	avg	1.29	1.42	0.91	1.23	4.26
	stder	0.25	0.21	0.24	0.17	0.68
Tukey’s post hoc test	WT v. Q97	0.86	<0.001	<0.001	0.22	<0.05
	WT v. Q31	0.23	0.83	1.00	1.00	0.87
	Q31 v. Q97	0.24	<0.001	<0.001	0.17	<0.01

### MSN passive membrane and action potential properties are altered in both Q31 and Q97 mice

**Fig 2:**
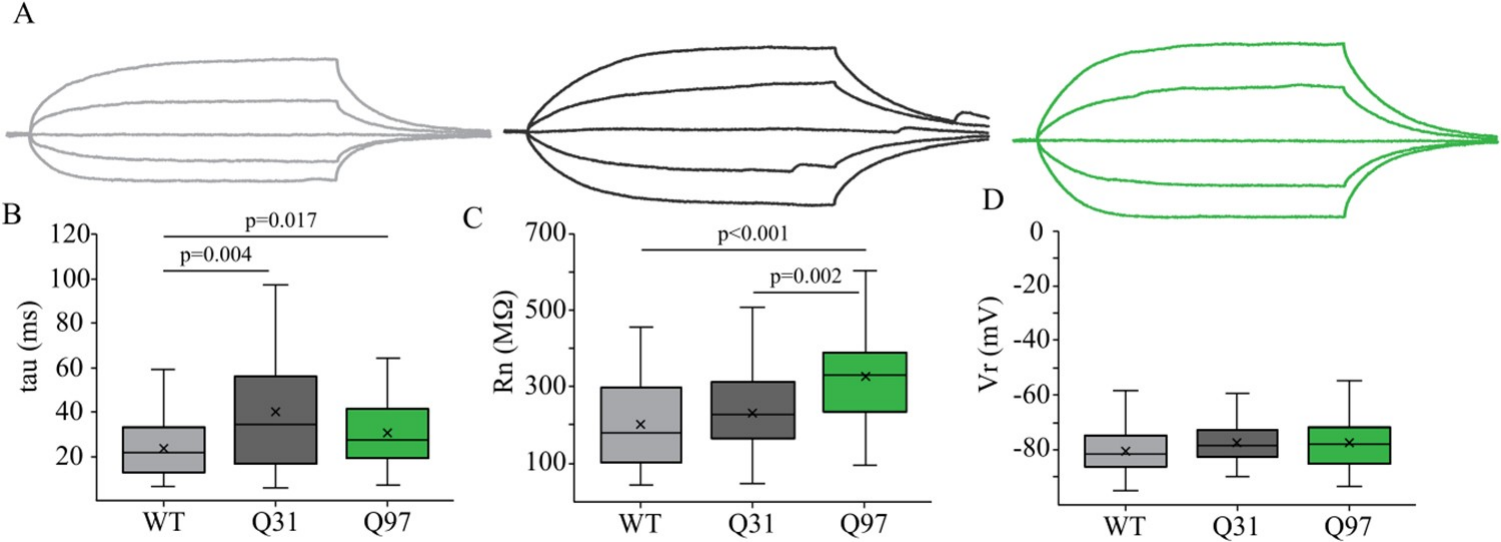
Passive membrane properties of MSNs across genotypes. (a) Membrane voltage responses to a series of 200ms hyperpolarizing and depolarizing current steps of representative MSNs from WT (light grey), Q31 (dark grey) and Q97 (green) mice. Scale bar: 10mV/20ms. (b) Longer membrane time constant (tau) in Q31 and Q97 compared to WT MSNs (WT, n = 54; Q31, n = 30; Q97, n = 60; Kruskal-Wallis test: WT v. Q31, p = 0.004; WT v. Q97, p = 0.017). (c) Significantly higher input resistance of MSNs in Q97 compared to both WT and Q31 mice (WT, n = 60; Q31, n = 34; Q97, n = 77; Kruskal-Wallis test: WT v. Q97, p<0.001; Q31 v. Q97, p = 0.002). (d) Resting membrane potential did not differ between the three genotypes. (WT, n = 58; Q31, n = 34; Q97, n = 82; ANOVA, p = 0.127).

**Fig 3:**
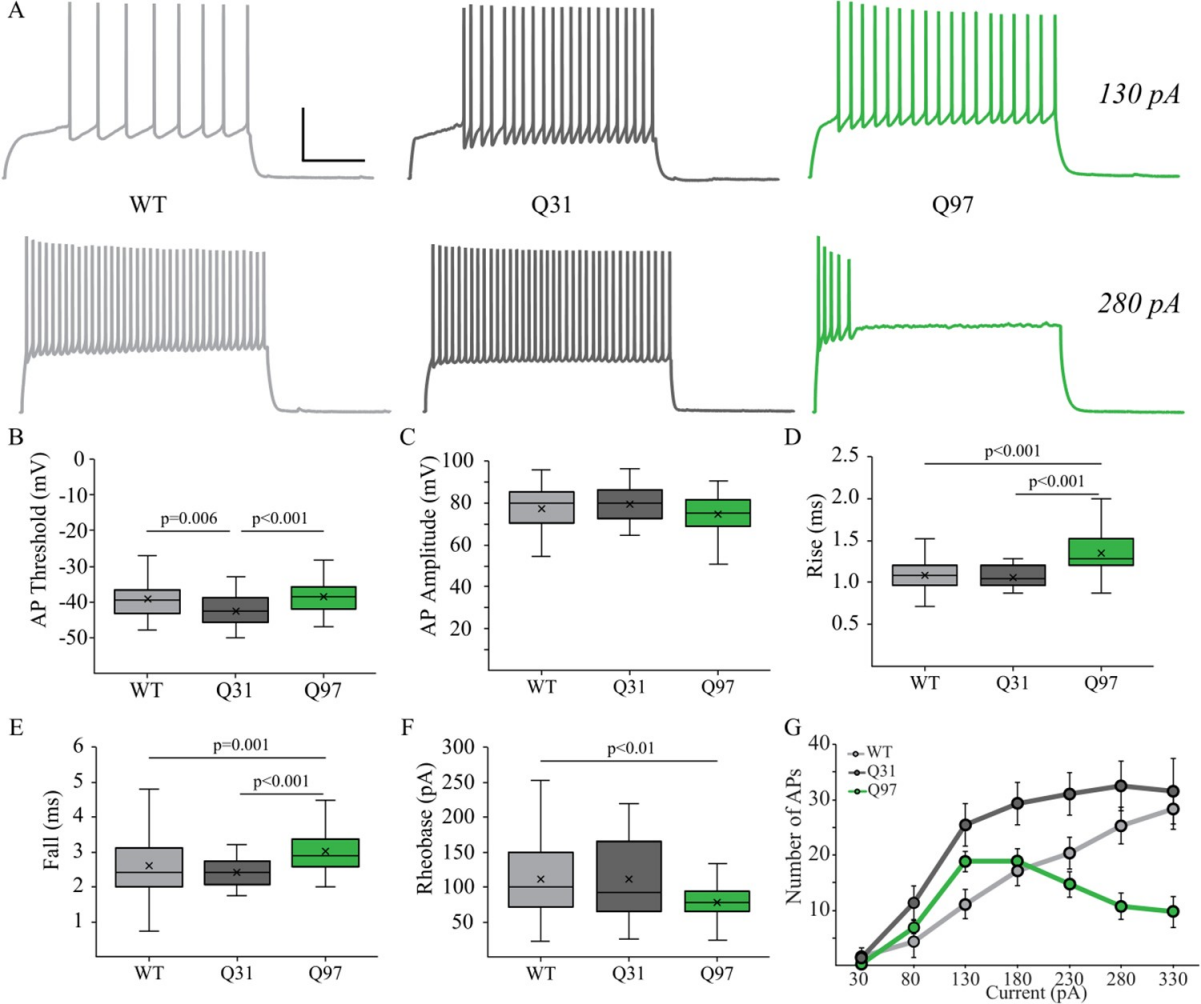
Single and repetitive action potential (AP) properties of MSNs across genotypes. (a) Membrane voltage responses to 2s 130 pA (top) and 280pA (bottom) depolarizing current steps of representative MSNs from WT, Q31 and Q97 mice. Scale bar: 40mV/200ms. (b) AP threshold was significantly lower in Q31 MSNs compared to the other two groups but did not differ between WT and Q97 MSNs (WT, n = 46; Q31, n = 34; Q97, n = 67; Tukey’s test: WT v. Q31, p = 0.006; Q31 v. Q97, p<0.001). (c) AP amplitude did not differ between the three groups (WT, n = 47; Q31, n = 33; Q97, n = 67; ANOVA, p = 0.059). (d, e) the rise and fall times of APs were significantly longer in Q97 MSNs compared to both WT and Q31 MSNs (Rise: WT, n = 44; Q31, n = 33; Q97, n = 65; Kruskal-Wallis test: WT v. Q97, p<0.001; Q31 v. Q97, p<0.001; Fall: WT, n = 45; Q31, n = 31; Q97, n = 63; Kruskal-Wallis test: WT V. Q97, p = 0.001; Q31 v. Q97, p<0.001). (f) Rheobase was significantly lower in Q97 MSNs compared to WT MSNs (WT, n = 48; Q31, n = 33; Q97, n = 63; Tukey’s test: WT v. Q97, p<0.001). (g) Mean *f-I* curves for MSNs of each genotype. (*WT and Q31, **WT and Q97, and #Q31 and Q97; 130 pA, *p<0.01; 180 pA, *p = 0.01, #p<0.05; 230 pA, *p<0.05, #p<0.001; 280 pA, **p<0.01, #p,0.001; 330 pA, **p<0.01, #p<0.01; p-values from *posthoc* Tukey tests).

Significant alterations to basic electrophysiological properties of MSNs within the dorsolateral striatum have consistently been reported in multiple mouse models of HD [28,29,31–34,53]. Here, we employed whole-cell patch clamp recordings of MSNs in the dorsolateral striatum of WT, Q31, and Q97 mice to characterize these properties. Passive membrane and action potential properties did not differ between males and females within each genotype (Tau: F_(1,128)_ = 0.7486, p = 0.3885; Rn: F_(1,166)_ = 0.5726, p = 0.4503; Vr: F_(1,166)_ = 0.0001, p = 0.9925; AP Threshold: F_(1,141)_ = 2.431, p = 0.1212; AP Amplitude: F_(1.141)_ = 0.3735, p = 0.5421; AP Rise: F_(1.136)_ = 0.1288, p = 0.0785; AP Fall: F_(1,133)_ = 2.012, p = 0.1584; Rheobase: F_(1,137)_ = 0.1133, p = 0.7370). As such, for electrophysiological and morphological analyses that follow, genotype groups include both male and female mice. MSNs in Q31 and Q97 mice exhibited significantly longer membrane time constant (tau) compared to WT neurons, while tau in Q31 and Q97 MSNs did not differ (Fig 2b). The input resistance of MSNs in Q97 mice was significantly higher compared to both WT and Q31 groups (Fig 2a and 2c). Resting membrane potential did not differ between the three genotypes (Fig 2d). Next, we assessed individual action potential parameters and firing frequency in MSNs from each genotype (Fig 3a). Action potential threshold was significantly hyperpolarized in Q31 MSNs compared to the other two groups (Fig 3b), but did not differ between WT and Q97 MSNs. The amplitude of single action potentials did not differ between the three groups (Fig 3c), but the rise and fall times of action potentials were significantly higher in Q97 MSNs compared to both WT and Q31 MSNs (Fig 3d and 3e). Rheobase was significantly lower in Q97 MSNs compared to WT, but did not differ between Q31 and Q97 MSNs (Fig 3f). A series of 2s depolarizing current steps revealed differences in evoked firing frequency between groups (Fig 3a and 3g). Firing frequency did not differ between male and female mice within each genotype so both sexes were grouped by genotype for firing frequency analysis (30 pA: F_(1,119)_ = 1.313, p = 0.2541; 80 pA: F_(1,119)_ = 2.107, p = 0.1492; 130 pA: F_(1,120)_ = 2.348, p = 0.1282; 180 pA: F_(1,109)_ = 1.039, p = 0.3103; 230 pA: F_(1,94)_ = 0.1722, p = 0.6791; 280 pA: F_(1,77)_ = 0.4615, p = 0.4991; F_(1,63)_ = 0.0903, p = 0.7648). Interestingly, firing frequency of MSNs from Q31 mice was greater than WT MSNs at 130–230 pA and both Q31 and WT MSNs exhibited higher firing rates than Q97 MSNs at 230–330 pA. At the highest amplitude depolarizing current steps (230-330pA) Q97 MSNs exhibited marked adaptation of firing, which was not seen in WT or Q31 MSNs (Fig 3a and 3g; Q97 vs WT = 130 pA: F_(2,120)_ = 6.64, p<0.01; 180 pA, F_(2,109)_ = 4.75, p = 0.01; 230 pA, F_(2,94)_ = 7.21, p = 0.001; 280 pA, F_(2,77)_ = 9.72, p<0.001; 330 pA, F_(2,63)_ = 7.80, p<0.001).

### Excitatory and inhibitory synaptic currents are altered in both Q31 and Q97 MSNs

**Fig 4:**
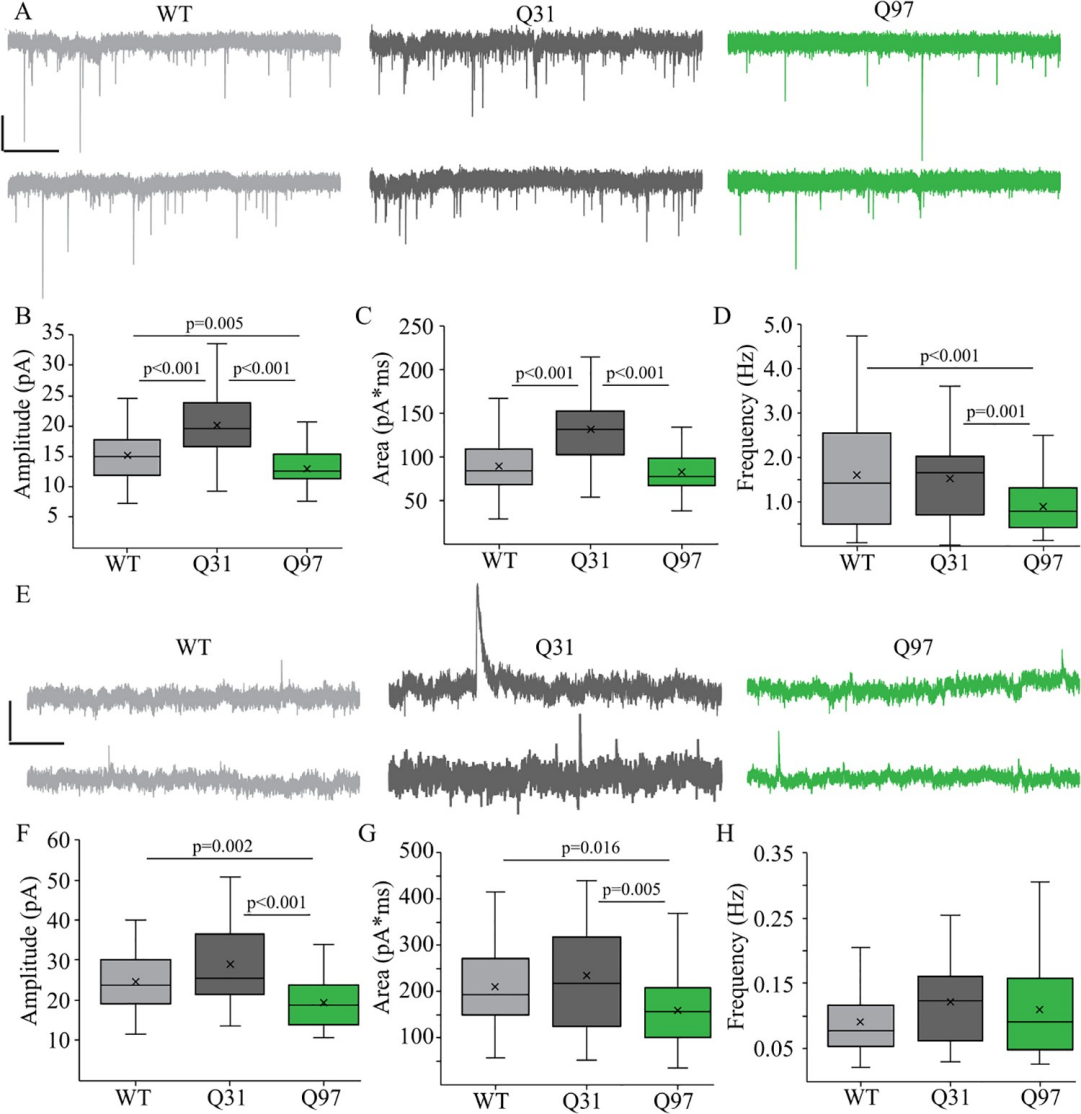
Excitatory and inhibitory synaptic current properties of MSNs across genotypes. (a) Representative spontaneous excitatory postsynaptic currents (EPSCs) recorded from MSNs from WT, Q31 and Q97 mice. (b) Mean amplitude of EPSCs, was significantly higher in Q31 than in WT MSNs, but lower in Q97 compared to WT MSNs (WT, n = 65; Q31, n = 38; Q97, n = 77; Tukey’s test: WT v. Q31, p<0.001; WT V. Q97, p = 0.005; Q31 v. Q97, p<0.001). (c) EPSC area was higher in Q31 MSNs compared to WT and Q97 MSNs (WT, n = 69; Q31, n = 38; Q97, n = 80; Kruskal-Wallis test: WT v. Q31, p<0.001; Q31 v. Q97, p<0.001). (d) Mean frequency of EPSCs was significantly lower in Q97 MSNs compared to both WT and Q31 MSNs (WT, n = 70; Q31, n = 40; Q97, n = 86; Kruskal-Wallis test: WT v. Q97, p = 0.002; Q31 v. Q97, p<0.001). (e) Representative spontaneous inhibitory postsynaptic currents (IPSCs) recorded from MSNs from WT, Q31 and Q97 mice. (f) Mean amplitude of IPSCs was higher in Q31 MSNs compared to WT and lower in Q97 MSNs compared to WT (WT, n = 42; Q31, n = 34; Q97, n = 60; Kruskal-Wallis test: WT v. Q97, p = 0.002; Q31 v. Q97, p<0.001). (g) Mean IPSC area was significantly lower in Q97 MSNs compared to both WT and Q31 MSNs (WT, n = 40; Q31, n = 29; Q97, n = 58; Kruskal-Wallis test: WT v. Q97, p = 0.016; Q31 v. Q97, p = 0.005). (h) Mean IPSC frequency did not differ in MSNs from the 3 genotypes. (WT, n = 39; Q31, n = 32; Q97, n = 51; Kruskal-Wallis test: p = 0.18).

Excitatory and inhibitory synaptic currents response properties have consistently been reported to be altered in MSNs of mouse models of HD [29,32,53]. We compared both excitatory and inhibitory synaptic currents in MSNs from each of the genotypes (Fig 4). For synaptic analyses, male and female mice did not differ so sexes were grouped by genotype (EPSC: Amplitude, F_(1,174)_ = 0.9038, p = 0.3431; Area, F_(1,181)_ = 2.386, p = 0.1242; Frequency, F_(1,179)_ = 0.002, p = 0.969; IPSC: Amplitude, F_(1,130)_ = 1.883, p = 0.1724; Area, F_(1,112)_ = 0.9627, p = 0.3286); Frequency, F_(1,113)_ = 0.1027, p = 0.7492). The mean amplitude of excitatory postsynaptic currents (EPSCs), was significantly higher in Q31 than in WT MSNs, but lower in Q97 compared to WT MSNs (Fig 4b). Mean EPSC area was higher in Q31 MSNs compared to WT and Q97 (Fig 4c). Finally, the mean frequency of EPSCs was significantly lower in Q97 MSNs compared to both WT and Q31 MSNs (Fig 4d). The mean amplitude of inhibitory synaptic currents (IPSCs) was lower in Q97 MSNs compared to WT and Q31 (Fig 4f). IPSC area was significantly lower in Q97 MSNs compared to both WT and Q31 cells (Fig 4g). Finally, IPSC frequency did not differ in MSNs from the 3 genotypes (Fig 4h).

### Reduced dendritic length in Q31 -but not Q97- MSNs compared to WT

**Fig 5:**
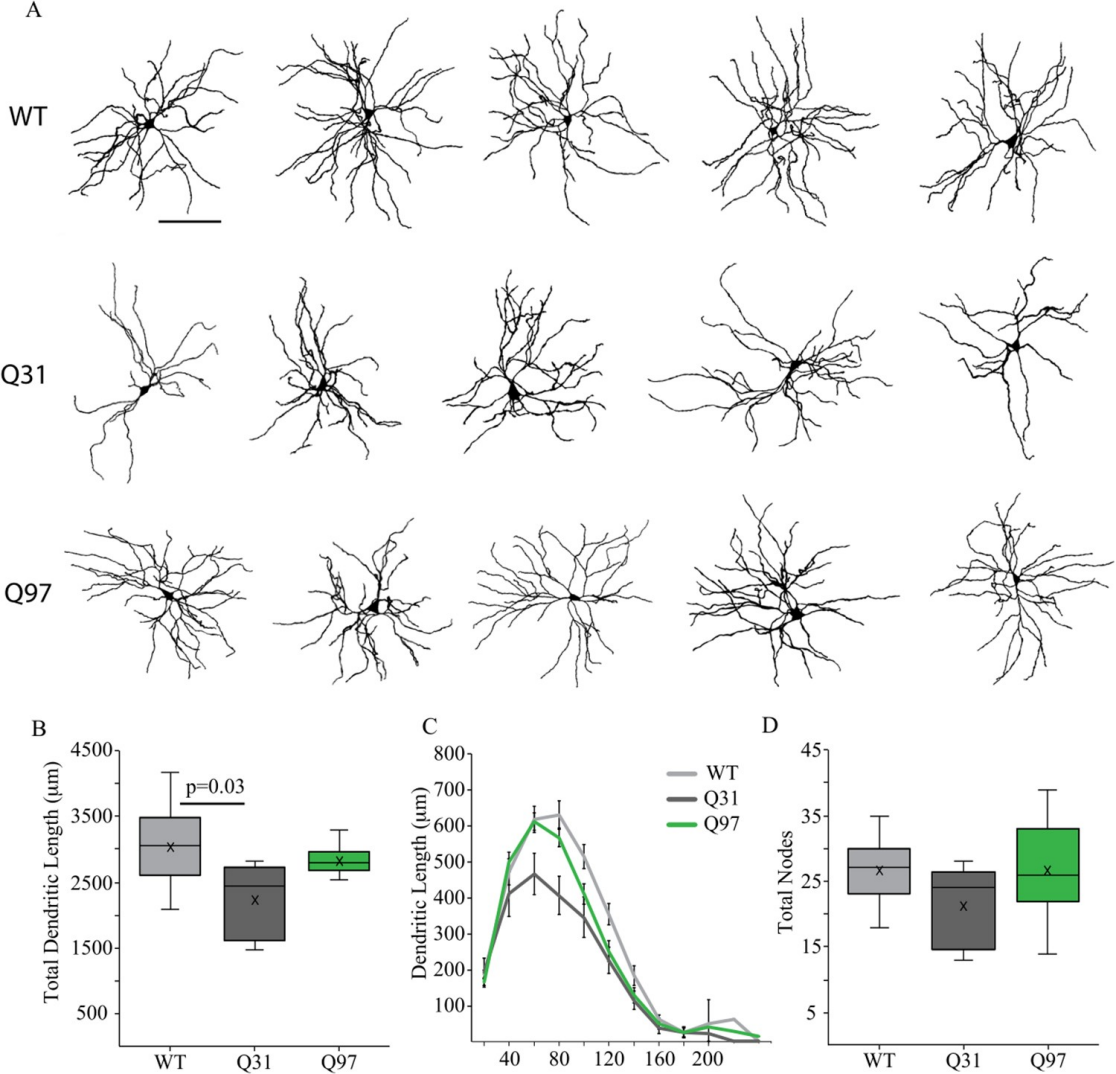
Dendritic morphology of MSNs across genotypes. (a) Representative reconstructions of the dendritic arbors of MSNs from WT, Q31 and Q97 mice (Scale bar: 100μm). (b) Lower mean total dendritic length of Q31 MSNs compared to WT and Q97 groups (WT, n = 16; Q31, n = 5; Q97, n = 15; Kruskal-Wallis test: WT v. Q31, p = 0.03). (c) Sholl analysis demonstrating that the reduction in Q31 dendritic length is statistically significant in the region 80–120 μm from the cell soma (*WT and Q31, **WT and Q97, and #Q31 and Q97; 80 μm, *p<0.01, #p<0.05; 100 μm, *p<0.05, **p<0.05; 120 μm, **p<0.05; p-values from *posthoc* Tukey tests). (d) Mean number of dendritic nodes (branch points), did not differ in MSNs from the 3 genotypes. (WT, n = 16; Q31, n = 5; Q97, n = 15; Kruskal-Wallis test: p = 0.25).

Dendritic topology of MSNs was assessed using confocal microscopy of neurons filled during whole-cell patch clamp recordings and reconstruction with NeuronStudio software (Fig 5a). For morphological analyses, male and female mice were grouped by genotype as there were no significant sex differences (Dendritic length: F_(1,31)_ = 2.568, p = 0.1192; Dendritic nodes: F_(1,33)_ = 0.0329, p = 0.8572). Total dendritic length was significantly different between groups, with lower mean dendritic length of Q31 MSNs compared to WT (Fig 5b). Sholl analysis of dendritic length revealed that the reduction in Q31 dendritic length was statistically significant in the region 60–120 μm from the cell soma (Fig 5c). By contrast the number of dendritic nodes (branch points), did not differ between genotypes (Fig 5d).

### Dendritic spine density and distribution do not differ between MSNs from WT, Q31, and Q97 mice

**Fig 6:**
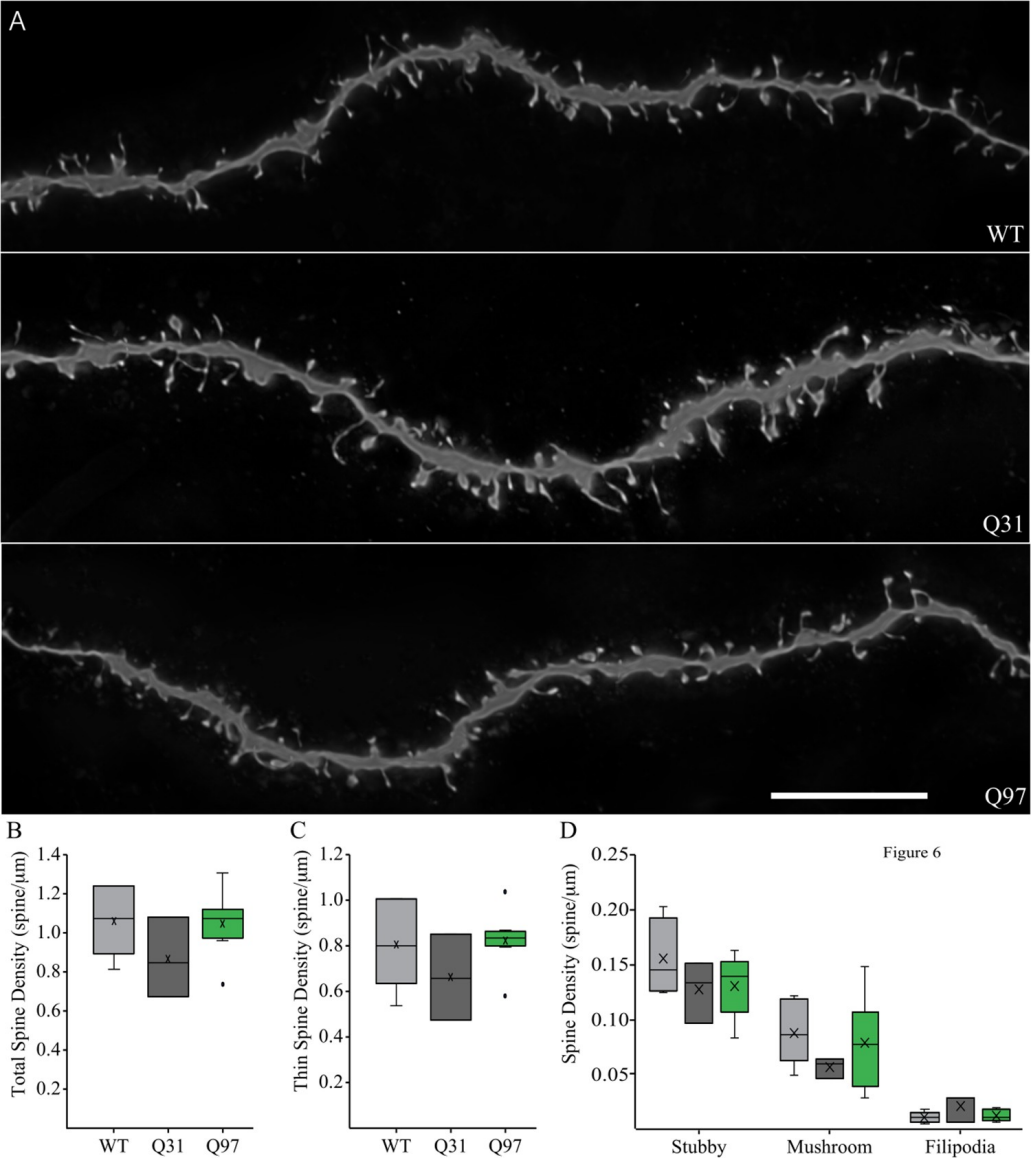
Dendritic spines of MSNs across genotypes. (a) Confocal image of WT, Q31, and Q97 MSN dendrites showing dendritic spines. Scale bar: 10 μm. (b) Mean density of dendritic spines did not differ significantly on MSNs between genotypes (WT, n = 6; Q31, n = 3; Q97, n = 8; ANOVA, p = 0.27) (c) Mean density of thin and of (d) stubby, mushroom, and filopodia spines did not differ between genotypes (WT, n = 6; Q31, n = 3; Q97, n = 8; ANOVA, thin, p = 0.35; stubby, p = 0.26; mushroom, p = 0.44; Kruskal-Wallis test: filipodia, p = 0.49).

Three dendrites were selected from each neuron for high resolution confocal imaging of dendritic spines (Fig 6a). Spine density did not differ between male and female animals, so sexes were combined by genotype for spine analyses (Spines: Total, F_(1,11)_ = 3.762, p = 0.0784; Thin, F_(1,11)_ = 4.111, p = 0.0675; Stubby, F_(1,11)_ = 0.4912, p = 0.4980; Mushroom, F_(1,11)_ = 0.013, p = 0.9122; Filopodia, F_(1,11)_ = 1.236, p = 0.290). The density of dendritic spines did not differ significantly on MSNs between genotypes (Fig 6b). Individual spine sub-types were classified using established criteria: thin (head diameter ≤ 0.6 μm), mushroom (head diameter > 0.6 μm), stubby (spines lacking a neck), or filopodia (length > 3 μm; [50,52]). The density of the most abundant subtype, thin spines, was similar between WT, Q31, and Q97 dendrites (Fig 6c). Similarly, the density of stubby, mushroom, and filopodia spines did not differ between genotypes (Fig 6d).

## Discussion

The N17 domain of the Huntingtin gene, which regulates nuclear export of mHtt has been suggested to play a pivotal role in mutant Htt aggregation and dysfunction [37–46]. Earlier studies in animal models of HD have explored how deletion of the N17 domain (ΔN17) influences mHtt nuclear inclusions and the manifestation of HD-like phenotypes [47,48]. Here we sought to determine whether N17 deletion in BACHD and BACWT mice results in exacerbation of structural and functional changes to dorsolateral striatal MSNs compared to WT. The key findings of the study were that: Q97 mice exhibit impaired gait and righting reflex but normal tail suspension reflex and normal coat while Q31 mice do not differ from WT; with a few exceptions, intrinsic membrane and action potential are altered in MSNs from Q97 but not Q31 mice; excitatory and inhibitory synaptic currents exhibit higher amplitudes Q31 but not Q97 MSNs while excitatory synaptic currents occurred at greater frequency in Q97 than in WT and Q31, and; there is a reduced total dendritic length in Q31 -but not Q97- MSNs compared to WT, while spine density and number did not differ between the three groups.

### Motor phenotypes and weight reduction in Q97 but not Q31mice

Weight loss and reduction in volume of the striatum, motor cortex and other brain structures occur during HD progression [7–12,56]. Some HD models (e.g. R6/2 and Q175^+/-^) exhibit weight loss but other models (e.g. YAC128 and BACHD) actually weigh more, not less, than WT littermates [31,57–62]. By contrast to these differences in weight loss phenotypes, HD mouse models consistently exhibit reductions in brain weight over the course of disease progression [31,57–62]. Q97 mice have previously been reported to weigh more than WT mice at 2–6 months of age, by 9 months of age they exhibit significant weight loss [48] reminiscent of models such as the R6/2 and Q175^+/-^ [31, 57]. In the present study, male Q97 mice weighed significantly less than both Q31 and WT males whereas female Q97 mice did not differ from either of the other cohorts at 9 months of age. By contrast, both female and male Q97 mice showed significantly lower brain weight. In their initial assessment of ΔN17-Q97 mice, Gu et al., (2015) reported major motor phenotypes -including chorea/dystonia-like movements observed during gait assessment beginning at 7 months of age that seemed reminiscent of choreiform movements seen in HD and never before reported in mouse models of the disease. The health and motor phenotypes observed here were markedly less pronounced compared to those reported earlier [48]. Thus, nine-month-old Q97 (but not Q31) mice in the present study exhibited modest dysfunctional motor phenotypes but had normal coats and did not exhibit the marked chorea/dystonia phenotype reported previously in 7-10-month-old Q97 mice [48]. This difference in motor phenotype is puzzling, since it cannot be accounted for by a difference in age or in genotype of the experimental subjects.

### Changes to Q97 MSNs intrinsic membrane properties are consistent with other HD models

Electrophysiological assessment of MSNs in the R6/2 and Q175 HD mouse models have established consistent changes to intrinsic membrane properties including increased input resistance, reduced rheobase, altered action potential kinetics and decreased excitatory postsynaptic current frequency [28–33,53]. Consistent with these previous findings, MSNs in the striatum of Q97 mice exhibited significantly increased input resistance, reduced rheobase, and reduced EPSC frequency. Similarly, markedly increased adaptation of action potentials firing at higher current steps suggests a reduced dynamic range of Q97 MSNs that has also been observed in the symptomatic R6/2 model [28]. This increase in adaptation if present in vivo would be expected to alter network behavior within the striatum and in the cortico-striatal-thalamo-cortical circuit. As with alterations to MSNs seen in other HD models, changes to the intrinsic membrane properties of MSNs in Q97 mice are likely due to changes in ion channel expression. Indeed, in the R6/2 and Q175 mouse models, reductions in conductance of inward and outward rectifying potassium channels has been reported [63–66]. In the Q175, proteomic analyses have also revealed reductions in the expression of potassium channels including the inward rectifying potassium channel, Kir2.3 [67]. It is not known whether MSNs in BACHD mice undergo similar changes to potassium channel conductance and expression, but gene expression analyses of the BACHDΔN17 showed reduction in the expression of multiple potassium channels including: Kir2.3, Kv11.1, Kv8.1, and TREK-1 [48]. If the BACHD does indeed lack changes to potassium channel expression, it is possible that the deletion of the N17 domain and subsequent nuclear inclusion of mHtt species underlie changes to gene expression contributing to a reduction in potassium channels and changes to passive properties reported here in the BACHDΔN17, but not BACHD. Further genetic and proteomic comparison of BACHD and BACHDΔN17 mice are necessary to understand how the N17 domain may contribute to abnormal gene expression in these mouse models of HD.

While an increase in input resistance and reduction in rheobase may suggest that MSNs in the Q97 are more excitable.

### Structural properties of Q97 neurons did not differ from WT

Previous studies have reported alterations to dendritic arbors and/or loss of dendritic spines in striatal MSNs in the R6/2, Q175, and BACHD models [28,32,34,53]. In the BACHD, MSN dendritic arbors do not differ from WT but there is a significant reduction in the density of dendritic spines [34]. Here, we report that dendritic length and dendritic arbor complexity did not differ between WT and Q97 MSNs and there were also no changes in dendritic spine density in Q97 MSNs. This raises the question of how the deletion of the N17 domain may ameliorate spine loss seen in BACHD mice. The Huntingtin protein is known interact with cytoskeletal elements within the cytoplasm of neurons and proteins vital for anterograde and retrograde transport [68–73]. Interestingly, a critical interaction of Huntingtin is with the cytoskeletal element α-actinin [74], which localizes to the dendritic spine and is critical for actin organization, spine morphology, and PSD assembly [75]. It is possible that cytoplasmic mHtt is able to interact with this complex and cause the loss of spines mediated by spine cytoskeletal disorganization in the BACHD model. If this were the case, in the BACHΔN17, sequestration of mHtt to the nucleus may reduce cytoplasmic mHtt levels limiting the interaction of mHtt and α-actinin, and thus preventing the development of the spine pathology. Further work on the BACHDΔN17 will be necessary to determine how the deletion of the N17 domain affects mHtt:protein interactions and if this deletion also affects the protein interactions of WT Htt which could possibly explain the changes to morphology reported here in Q31 MSNs.

### Changes to Q31 MSN physiological and structural features

Similar to Gu et al., (2015) the body and brain weights of Q31 mice were normal; however, to our surprise MSNs from these mice exhibited several significant physiological and morphological differences from WT and Q97 neurons. Deletion of the N17 domain of the mHtt gene in BACHD mice results in physiological phenotypes similar to those reported in other models, but not seen in the BACHD model alone; is it possible that the deletion of N17 may also affect how the *normal* Htt protein influences physiological and structural properties of MSNs? Our data suggests that the answer to this question may be yes. Q31 MSNs exhibited significant alterations that were not seen in MSNs from WT or Q97 mice including: a longer time constant, hyperpolarized action potential threshold, higher action potential firing frequency, increased amplitude EPSCs and IPSCs and reduced dendritic lengths. Thus, our data suggests that deletion of N17 from WT Htt results in changes to MSNs which may be a consequence of nuclear inclusion of the WT Htt protein. Htt has been demonstrated to interact with various transcription factors and regulators and thus to influence gene expression [76–78]. If the normal distribution of Htt is disrupted by the deletion of N17, it is possible that nuclear inclusion of Htt disrupts expression of genes responsible for ion channels and structural proteins that influence neuron function and structure. While it is hypothesized that changes to channel gene expression underlie many of the physiological abnormalities reported in mouse models of HD, it is clear that the deletion of the N17 domain results in changes to gene expression in BACHD mice [48], the Q31 (BACWTΔN17) model may also experience changes at the genomic level and future studies of this model may be useful in understanding how the various domains of the Htt gene influence transcription.

### Implications of these findings for N17 in HD

Our findings in the Q97 (BACHDΔN17) mouse suggest a role for the N17 domain in the presentation of HD-like phenotypes seen in other mouse models of HD. While many of our electrophysiological findings mirror studies in other models, there are some differences which indicate the importance of the N17 domain and potentially mHtt cytoplasmic localization in pathogenesis. First, BACHDΔN17, but not BACHD mice, mirror other mouse models with a decrease in body weight at advanced ages and may be related to the nuclear inclusion of mHtt species in the BACHDΔN17 resulting in transcriptional changes. Second, the BACHDΔN17 exhibited changes to physiological properties of MSNs not seen in the BACHD which may be related to transcriptional down regulation of potassium channels reported in the BACHDΔN17, but not seen in BACHD mice. Third, the preservation of dendritic spines in MSNs of Q97 mice indicates the nuclear inclusion of mHtt may prevent dysfunctional association of mHtt with cytoskeletal elements in this model, preventing the manifestation of the spine loss phenotype. Finally, the lack of prominent differences in D1- and D2-MSN sub-populations suggests that the deletion of the N17 domain may partially normalize the effects that cytoplasmic mHtt has on the differential pathophysiology and phenotypes seen in MSN sub-types. Together, our findings are consistent with the notion that N17 plays a role in modifying the functions of both WT Htt and mutant Htt in this mouse model of HD.
