# Correction: Structural and functional features of medium spiny neurons in the BACHDΔN17 mouse model of Huntington’s Disease

**DOI:** 10.1371/journal.pone.0327837

**Published:** 2025-07-10

**Authors:** Joseph Goodliffe, Anastasia Rubakovic, Wayne Chang, Dhruba Pathak, Jennifer Luebke

This Correction brings the article [1] into compliance with PLOS’ Data Availability policy, provides further information on the source of the mouse constructs used in [1], and addresses errors identified post-publication.

## Data availability

The repository listed in the Data Availability statement of this article [1] includes neuronal morphology data but underlying data for the other results reported in the article are not included. The underlying data for the entire original raw dataset, including behavioral, electrophysiology and neuronal morphology data, are now uploaded to a data repository and can be found at [2].

The new Data Availability statement is: All data files are deposited in Dryad Digital Repository. Doi: https://doi.org/10.5061/dryad.v6wwpzh42.

## Sourcing of mice constructs

The Drd2eGFPxQ31deltaN17 mouse line (referred to in the article as BACWTΔN17 (Q31)) was obtained through The Jackson Laboratory [3] via a special arrangement with The CHDI Foundation [4], and mouse tails were sent to an external laboratory for confirmatory genotyping. Readers wishing to request access to the mouse line should contact The Jackson Laboratory [3] or CHDI Foundation [4].

## Figure and text errors

With this Correction, the corresponding author provides revised versions of Figs 1 and 5 to address errors in Fig 1A–B and Fig 5D, and errors in the figure legends of Figs 1–3. Please see the complete, correct Fig 1–3 captions below. Specifically,

**Fig 1:**
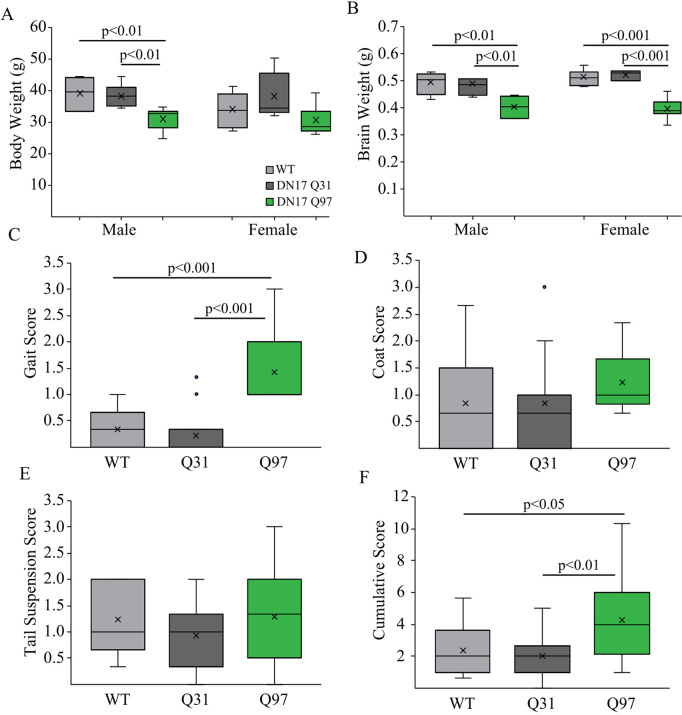
Body and brain weight and motor function in WT, Q31 and Q97 mice. (a) Male Q97 mice weighed significantly less than both Q31 and WT mice, while there was no difference between genotypes in female mice (Male: WT, n = 6; Q31, n = 10; Q97, n = 7; F_(2,22)_ = 8.66, p = 0.002; Female: WT, n = 7; Q31, n = 5; Q97, n = 7; F_(2,18)_ = 2.47, p = 0.116). (b) In both males and females, brain weight was significantly lower in Q97 compared to Q31 and WT subjects (which did not differ, Male: WT, n = 6; Q31, n = 10; Q97, n = 6; F_(2,21)_ = 9.45, p = 0.001; Female: WT, n = 7; Q31, n = 5; Q97, n = 7; F_(2,18)_ = 33.2, p < 0.001;). (c) Q97 mice exhibited significantly impaired gait (WT, n = 11; Q31 = 15; Q97 = 11). (d) Coat quality did not differ between groups (WT, n = 13; Q31 = 15; Q97 = 13). (e) Tail suspension score did not differ between groups (WT, n = 13; Q31, n = 15; Q97, n = 14). (f) Cumulative score of animal performance in the motor tasks plus coat quality assessment revealed significant impairment in Q97 mice (WT, n = 13; Q31, n = 15; Q97, n = 14; p-values from *posthoc* Tukey tests).

**Fig 2:**
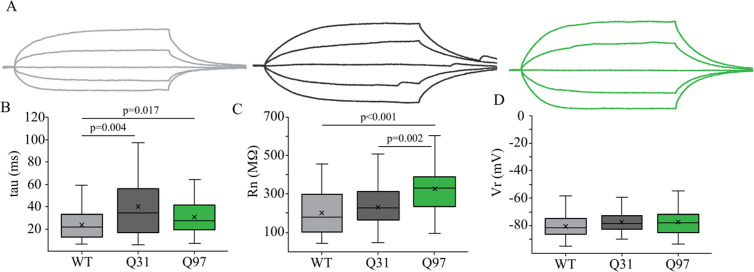
Passive membrane properties of MSNs across genotypes. (a) Membrane voltage responses to a series of 200ms hyperpolarizing and depolarizing current steps of representative MSNs from WT (light grey), Q31 (dark grey) and Q97 (green) mice. Scale bar: 10mV/20ms. (b) Longer membrane time constant (tau) in Q31 and Q97 compared to WT MSNs (WT, n = 54; Q31, n = 30; Q97, n = 64; Kruskal-Wallis test: WT v. Q31, p = 0.004; WT v. Q97, p = 0.017). (c) Significantly higher input resistance of MSNs in Q97 compared to both WT and Q31 mice (WT, n = 60; Q31, n = 34; Q97, n = 77; Kruskal-Wallis test: WT v. Q97, p < 0.001; Q31 v. Q97, p = 0.002). (d) Resting membrane potential did not differ between the three genotypes. (WT, n = 58; Q31, n = 34; Q97, n = 82; ANOVA, p = 0.127).

**Fig 3:**
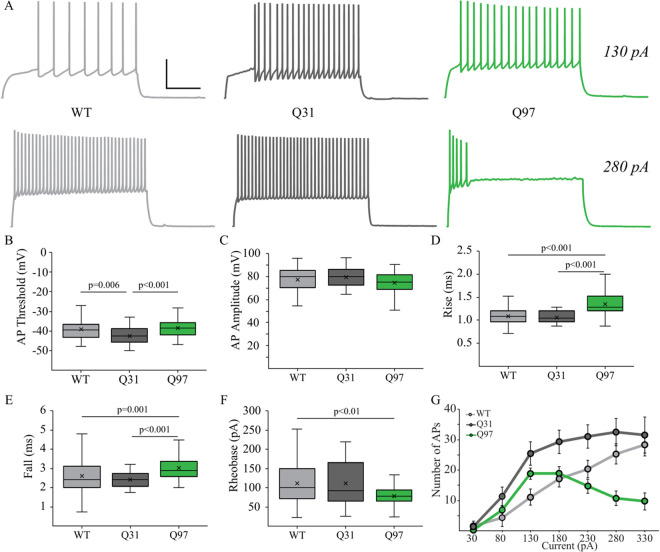
Single and repetitive action potential (AP) properties of MSNs across genotypes. (a) Membrane voltage responses to 2s 130 pA (top) and 280pA (bottom) depolarizing current steps of representative MSNs from WT, Q31 and Q97 mice. Scale bar: 40mV/200ms. (b) AP threshold was significantly lower in Q31 MSNs compared to the other two groups but did not differ between WT and Q97 MSNs (WT, n = 46; Q31, n = 34; Q97, n = 67; Tukey’s test: WT v. Q31, p = 0.006; Q31 v. Q97, p < 0.001). (c) AP amplitude did not differ between the three groups (WT, n = 47; Q31, n = 33; Q97, n = 67; ANOVA, p = 0.059). (d, e) the rise and fall times of APs were significantly longer in Q97 MSNs compared to both WT and Q31 MSNs (Rise: WT, n = 44; Q31, n = 33; Q97, n = 65; Kruskal-Wallis test: WT v. Q97, p < 0.001; Q31 v. Q97, p < 0.001; Fall: WT, n = 44; Q31, n = 31; Q97, n = 63; Kruskal-Wallis test: WT V. Q97, p = 0.001; Q31 v. Q97, p < 0.001). (f) Rheobase was significantly lower in Q97 MSNs compared to WT MSNs (WT, n = 48; Q31, n = 33; Q97, n = 63; Tukey’s test: WT v. Q97, p < 0.001). (g) Mean *f-I* curves for MSNs of each genotype. (*WT and Q31, **WT and Q97, and #Q31 and Q97; 130 pA, *p < 0.01; 180 pA, *p = 0.01, #p < 0.05; 230 pA, *p < 0.05, #p < 0.001; 280 pA, **p < 0.01, #p,0.001; 330 pA, **p < 0.01, #p < 0.01; p-values from *posthoc* Tukey tests).

**Fig 5:**
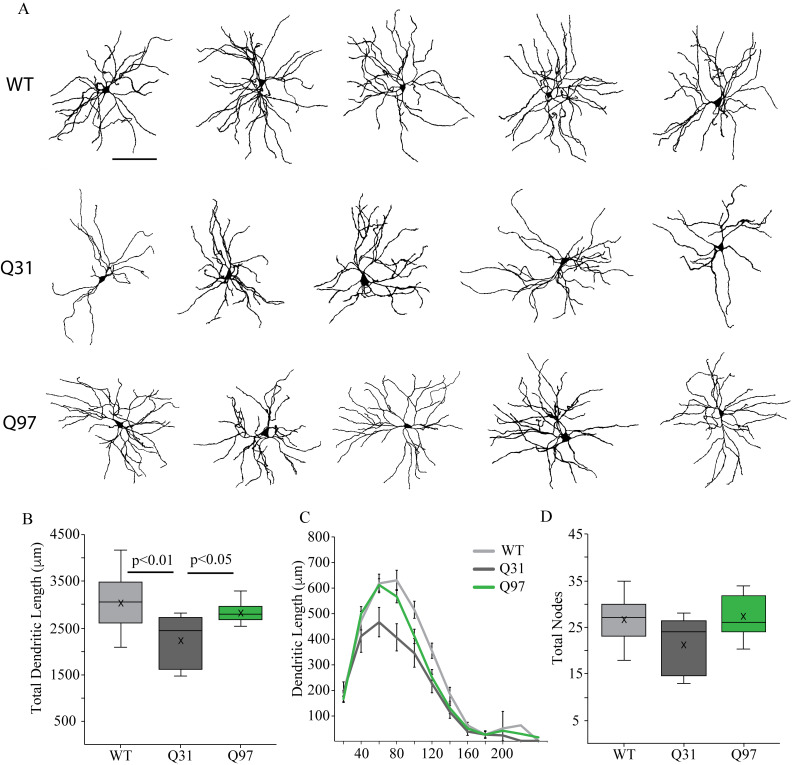
Dendritic morphology of MSNs across genotypes. (a) Representative reconstructions of the dendritic arbors of MSNs from WT, Q31 and Q97 mice (Scale bar: 100μm). (b) Lower mean total dendritic length of Q31 MSNs compared to WT and Q97 groups (WT, n = 16; Q31, n = 5; Q97, n = 15; Kruskal-Wallis test: WT v. Q31, p = 0.03). (c) Sholl analysis demonstrating that the reduction in Q31 dendritic length is statistically significant in the region 80–120 μm from the cell soma (*WT and Q31, **WT and Q97, and #Q31 and Q97; 80 μm, *p < 0.01, #p < 0.05; 100 μm, *p < 0.05, **p < 0.05; 120 μm, **p < 0.05; p-values from *posthoc* Tukey tests). (d) Mean number of dendritic nodes (branch points), did not differ in MSNs from the 3 genotypes. (WT, n = 16; Q31, n = 5; Q97, n = 15; Kruskal-Wallis test: p = 0.25).

- There were errors in the dataset used to calculate the box-plot values for body weight and brain weight, respectively, for WT male mice in Fig 1A-B, which are addressed in the revised Fig 1. The authors confirm this error does not affect the statistical analyses reported in Fig 1 or the overall findings reported for Fig 1 in the Results section.
- There were errors in the dataset used to calculate the Total Nodes box-plot values for Q97 mice in Fig 5D, which are addressed in the revised Fig 5. The authors stated that outlier statistics were performed on the dataset for total nodes which identified four cells as outliers in the Q97 group; these data were removed bringing the sample size down from 19 to 15, but this was not reflected in the original dataset. The authors confirm this error does not affect the statistical analyses reported in the caption for Fig 5D or the overall findings reported for Fig 5 in the Results section.
- In the Fig 1D legend text, the reported n value for coat quality in Q97 mice is incorrect and should be 13.
- In the Fig 2B legend text, the reported n value for Tau in Q97 mice is incorrect and should be 64.
- In the Fig 3E legend text, the reported n value for Fall in WT mice is incorrect and should be 44.

Additionally, there is an error in the first sentence of the Statistical analyses subsection of the Materials and methods in [1] which states that GraphPad was used to perform statistical analyses of empirical data. The first author stated that this is incorrect and MATLAB was used for all statistics reported in [1]. These underlying data are included in the Electrophysiology file of the dataset deposited at the above repository. The correct sentence is: Statistical analyses of empirical data were performed using MATLAB.

The authors confirm that the issues reported above do not affect the results and conclusions reported in [1]. The version history of the data deposits are detailed in the README files attached to each deposit in the Dryad Digital Repository.
